# Metagenomic analysis of endophytic bacteria in seed potato (*Solanum tuberosum*)

**DOI:** 10.1515/biol-2022-0897

**Published:** 2024-07-24

**Authors:** Rajapaksha Welhenage Piumi Madhushika Rajapaksha, Don Padmapriya Shantha Thilak Gunasekera Attanayaka, Kalaivani Vivehananthan, Dennis McNevin

**Affiliations:** Department of Biotechnology, Faculty of Agriculture & Plantation Management, Wayamba University of Sri Lanka, Makandura, Gonawila (NWP), Sri Lanka; Faculty of Science, Horizon Campus, Malabe, Sri Lanka; Department of Basic Sciences, Faculty of Health Sciences, The Open University of Sri Lanka, Nawala, Nugegoda, Sri Lanka; Centre for Forensic Science, School of Mathematical & Physical Sciences, Faculty of Science, University of Technology Sydney, Sydney, Australia

**Keywords:** ASVs, DADA2, firmicutes, 16s metagenomics, potato microbiome, NGS, proteobacteria

## Abstract

To date, the association of potato tuber microbiota is poorly understood. In this study, the endophytic bacterial flora of seed potato tubers was identified and the diversity of healthy and unhealthy tubers was compared. Metagenomic DNA extracted from healthy and unhealthy samples of seed potato tubers was used for the analysis of microbial communities. Next generation sequencing of the ∼460 bp v3–v4 region of the 16S rRNA gene was carried out using the Illumina Miseq platform. The data were analysed using the Divisive Amplicon Denoising Algorithm 2 pipeline. Sequence analysis of the potato metagenome identified amplicon sequence variants (ASVs) assigned to 745 different taxa belonging to eight Phyla: Firmicutes (46.2%), Proteobacteria (36.9%), Bacteroidetes (1.8%), Actinobacteria (0.1%), Tenericutes (0.005%), Saccharibacteria (0.003%), Verrucomicrobiota (0.003%), and Acidobacteria (0.001%). In healthy seed potato tubers, 55–99% of ASVs belonged to Firmicutes, including *Bacillus, Salinibacillus, Staphylococcus, Lysinibacillus, Paenibacillus*, and *Brevibacillus* genera within the taxonomic order Bacillales. However, in the visually unhealthy tubers, only 0.5–3.9% of ASVs belonged to Firmicutes while 84.1–97% of ASVs belonged to Proteobacteria. This study highlights that diverse bacterial communities colonize potato tubers, which contributes to the understanding of plant–microbe interactions and underscores the significance of metagenomic approaches in agricultural research.

## 1 Introduction

Potato (*Solanum tuberosum*) is one of the most important and widely grown crops in the world [1]. This crop provides vital sustenance for millions of people worldwide. However, the productivity and quality of potato crops are constantly threatened by various biotic and abiotic stressors, including pests, diseases, and environmental factors. Among these, bacterial pathogens pose a significant challenge to potato cultivation, leading to considerable yield losses and economic impacts. To protect the crop from biological invasions, a number of phytosanitary safeguards are adopted, including quarantine pathogen screening (*Ralstonia solanacearum*, *Clavibacter michiganensis*, *Pectobacterium caratovorum*, *Streptomyces scabies*) at the entry ports.

Endophytes represent an important source of microorganisms, which are taken up by plant roots and further colonize the plant interior [2]. They have beneficial effects on plant growth and development [3]. Endophytic bacteria, residing within the internal tissues of plants without causing any apparent harm, have been increasingly recognized for their potential role in promoting plant growth, enhancing stress tolerance, and providing protection against pathogens [4]. In the context of potato cultivation, understanding the diversity and functions of endophytic bacteria associated with seed potatoes is of particular interest, as seed potatoes serve as the primary source of planting material and can significantly influence the health and productivity of potato crops. Plant parts that are used as propagative materials are inevitably critical for successful crop production and the bacterial populations within them could play a major role in crop performance and production.

The plant microbiome is an integral part of the host and is increasingly recognized as playing a fundamental role in plant growth and health by forming complex co-associations with plants. Microbial biodiversity in seed potato tubers is poorly known and studied, and research has been limited to rhizosphere microorganisms. Analysis of the potato microflora would be important to understand its association with tuber health and to identify the biosecurity threats. Specially, in the case of imported seed potato, metagenomic analysis would provide a profile of bacterial flora, including endophytes as well as quarantine pathogens if available, revealing the ability for using metagenomics as a rapid screening tool.

Metagenomics is defined as the direct genetic analysis of genomes contained within environmental samples [5] and is the study of the collective genomes of the members of a microbial community. It involves isolating and analysing the genomes without culturing the organisms, thereby offering the opportunity to describe the plant’s diverse microbial inhabitants, many of which cannot yet be cultured [6]. Metagenomic studies have enhanced understanding of the roles of individual taxa in modulating plant physiology, colonization, and health. These approaches have helped to unravel the microbial diversity in various eco-habitats, where culture-based methods have failed because the majority of the microbes are unculturable and thereby go undetected using conventional methods. Recent developments in high-throughput next-generation sequencing technologies permit the investigation of endophytic microbiomes, facilitating the sequencing of a larger number of bacteria and in-depth analysis of bacterial communities as part of taxonomic, phylogenetic, and evolutionary studies [7]. Next-generation sequencing has dramatically accelerated the development of sequence-based metagenomics [8].

This study used the Divisive Amplicon Denoising Algorithm 2 (DADA2) pipeline to determine the composition of microbial communities. DADA2 offers the best sensitivity among the currently available metagenomic analysis pipelines [9] and it identifies variation at fine scale, minimizing the misinterpretation of sequence errors as biological variations [10]. The method was developed to resolve amplicon sequence variants (ASVs) from Illumina-scale amplicon data [11] and was explicitly intended to replace operational taxonomic units (OTUs) as the basic unit of biological variance. OTUs are clusters of reads that differ by less than a fixed sequence dissimilarity threshold, most commonly 3% [12]. ASV methods that are now available provide better resolution and accuracy than OTU methods [11] without imposing the arbitrary dissimilarity thresholds that define OTUs. Therefore, the metagenomic analysis of bacterial communities of seed potato tubers with the DADA2 pipeline would provide better understanding of the potato microbiome.

## 2 Materials and methods

### 2.1 Sample collection

**Figure 1:**
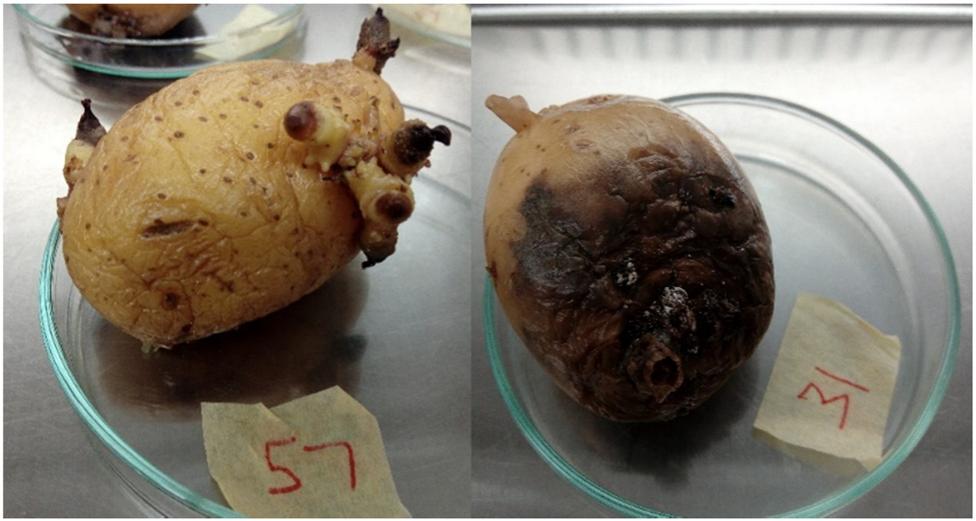
Healthy and unhealthy seed potato tubers collected at the entry ports.

Seed potato samples from 120 imported consignments were obtained from the plant quarantine station of the Department of Agriculture, Sri Lanka. The seed potato tubers were stored in a cold room below 4°C at the National Plant Quarantine Service at the time of sample collection. Of the 120 samples, 12 were randomly selected for metagenomic analysis of bacterial communities and these contained 9 visually healthy and 3 visually unhealthy and rotten samples (Figure 1). Tissues from two tubers of each sample were used for DNA extraction.

### 2.2 DNA extraction

The seed tubers were surface-sterilized with 10% NaOCl and then washed with distilled water prior to DNA extraction. Fifty grams (50 g) of each seed potato tuber (including both the peel and inside tissues) were crushed into a paste and transferred to a 50 mL falcon tube which contained 10 mL of autoclaved de-ionized water. The mixture was vortexed and filtered through a sterile gauze into another 50 mL falcon tube. The liquid extract was incubated for 5 min in a water bath adjusted to 95–100°C. The extract was then vortexed for 30 s and centrifuged at 12,400 × *g* for 10 min. The supernatant was removed and the pellet was washed with 3 mL of wash buffer (50 mM Tris-HCl, 5 mM EDTA, pH 8.0) and centrifuged again at 12,400 × *g* for 10 min. The supernatant was removed and an aliquot of 3 mL of lysis buffer (100 mM Tris-HCl, 100 mM EDTA, 1.5 M NaCl, pH 8.0) was added to the pellet, which was then incubated for 30–45 min with mixing at 10 min intervals. The homogenized mixture was centrifuged at 12,400 × *g* for 15 min. The resulting supernatant was divided into six 1.5 mL Eppendorf tubes, to each of which was added 150 µL of 3 M NaOAc and 150 µL of ice-cold isopropanol. Each tube was centrifuged at 12,400 × *g* for 15 min. The supernatant was removed and the pellet was washed with 70% ethanol, air-dried, and re-suspended in 25 µl of de-ionized water.

### 2.3 Seed potato microbiome analysis

Sequencing of the microbiome was performed by Macrogen (Korea). The V3 and V4 regions of the bacterial 16S rRNA gene were amplified using primers containing Illumina adapters following Illumina’s 16S Metagenomics Protocol (Part # 15044223 Rev. B). Briefly, the universal primers reported by Klindworth et al. [13] were used (16S Amplicon PCR Forward Primer = 5′TCGTCGGCAGCGTCAGATGTGTAT AAGAGACAGCCTACGGGNGGCWGCAG3′ and 16S Amplicon PCR Reverse Primer = 5′GTCTCGTGGGCTCGGAGATGTGTAT AAGAGACAGGACTACHVGGGTATCTAAT3′). Adapter-ligated fragments were then PCR-amplified and gel-purified. PCR was performed with the KAPA Hifi hot start PCR mix, and PCR clean-up was performed using AMPure XP Beads according to the 16S Metagenomics protocol. Amplicon libraries with a mean size of 460 bp were created. To verify the size of PCR-enriched fragments, the template size distribution was characterized on a 2100 Bioanalyzer (Agilent Technologies) using a DNA 1000 chip. The prepared libraries were quantified by qPCR according to the Illumina qPCR Quantification Protocol Guide. The library concentrations were adjusted to 4 µM and prepared for loading on a Miseq (Illumina), according to Illumina’s 16S Metagenomics Protocol. Samples were pooled, denatured, and loaded on the Miseq and sequenced with paired ends (2 × 300) using a MiSeq^®^ Reagent Kit v3 (600 cycle) (Part # 15044223 Rev. B).

V3-V4 primer trimming was performed using Cutadapt [14]. DADA2 [15] run on R software (version 3.5) was used for data processing and analyses. The sequences were filtered and trimmed to remove low-quality, short, and chimeric reads. Quality profiles of the forward and reverse reads were visualized using the plot Quality Profile command (Figure S1), and nucleotides from forward and reverse reads were trimmed based on the quality plot where forward reads less than 250 bp and reverse reads less than 200 bp were discarded. The fastq files were merged to form ASVs, which were then used to assign taxa with the Silva reference database (the ASV table generated from the DADA2 pipeline is included as Table S1). A user-defined mapping file was generated that contained the metadata. Alpha diversity (Shannon index) and beta diversity were visualized using principal coordinate analysis (PCoA) based on rarefied OTU counts using Phyloseq and Bray–Curtis distances between ASVs. All statistical analyses were accomplished with the vegan R package, DADA2 Pipeline, and Welch’s *t* test calculator (https://www.statology.org/welchs-t-test-calculator/). A *p* value < 0.05 was considered significant.

The sequences generated in this study were deposited in the National Center for Biotechnology Information Sequence Read Archive (http://www.ncbi.nlm.nih.gov/bioproject/PRJNA438178) under the accession numbers SRR24041404, SRR24041403, SRR24041402, SRR24041401, SRR24041400, SRR24041399, SRR24041398, SRR24041397, SRR24042715, SRR24042714, SRR24042713, and SRR24042712.

## 3 Results

### 3.1 Sequence data

Paired-end sequencing of libraries (MiSeq, Illumina) generated 500 Megabytes (500 Mb) of data. Quality filtering retained 2.05 million 16S rRNA gene sequence reads resulting in 1.38 million sequences distributed over 12 samples after chimeras were removed (Table 1), nearly all of which were ASVs (Table 2). There were 745 unique ASVs in total after accounting for those shared among the samples (Table S1). An average 170,901 filtered reads and 115,228 non-chimeric sequences were obtained from each sample (Table 2). The average amplicon size was 604 bp (Table 2). The ASVs were assigned to 745 different taxa by 7 taxonomic ranks. The 745 taxa belonged to Firmicutes (46.2%), Proteobacteria (36.9%), Bacteroidetes (1.8%), Actinobacteria (0.1%), Tenericutes (0.005%), Saccharibacteria (0.003%), Verrucomicrobiota (0.003%), and Acidobacteria (0.001%) (Table 2). About 13.6% of detected sequences were chloroplast sequences (Table 2).

**Table 1:** Summary of DNA extraction and amplicon library preparation metrics for seed potato samples

Sample		DNA concentration (ng/µl)	Amplicon library concentration (ng/µl)	Mean amplicon size (bp)	Number of filtered reads	Number of non-chimeric sequences
1.	01_13278	132.238	102.01	613	131,118	66,430
2.	16_50180	16.211	92.69	618	137,181	77,593
3.	31_12445	26.664	113.38	618	144,782	108,703
4.	47_11588	15.591	92.57	620	132,126	110,553
5.	49_51087	5.493	96.27	607	148,558	73,260
6.	57_51741	94.855	106.47	604	154,884	120,022
7.	66_51556	22.330	106.49	606	141,740	116,030
8.	82	24.405	101.39	613	129,390	70,823
9.	WYB36	1.797	36.13	568	237,028	153,529
10.	WYB27	41.672	55.84	596	251,553	123,462
11.	WYB21	70.545	61.22	594	228,310	220,031
12.	WYB71	3.654	56.66	595	214,142	142,296
Total					2,050,812	1,382,732
Average				604	170,901	115,228

**Table 2:** Distribution of ASVs across 12 seed potato samples in metagenomic analysis

Sample		Actinobacteria	Actinobacteria	Bacteroidetes	Firmicutes	Proteobacteria	Sacharibacteria	Tenericutes	Verrucomicrobiota	Chloroplasts	Total ASVs detected per sample	Percentage of ASVs (%)
1	01_13278	—	92	—	21,851	44,487	—	—	—	—	66,430	4.8
2	16_50180	—	—	2	76,834	145	—	—	—	606	77,587	5.6
3	31_12445	—	—	6	79,760	28,937	—	—	—	—	108,703	7.8
4	47_11588	—	2	29	110,123	352	—	—	—	31	110,537	7.9
5	49_51087	—	—	—	63,450	9,801	—	—	—	9	73,260	5.2
6	57_51741	—	—	—	109,387	10,632	—	—	—	3	120,018	8.6
7	66_51556	—	—	—	83,350	32,680	—	—	—	—	116,030	8.3
8	82	—	4	—	63,679	7,139	—	—	—	1	70,823	5.1
9	WYB36	15	393	332	10,495	30,910	15	—	—	177,871	220,031	15.9
10	WYB27	—	755	18,628	3,064	100,960	—	—	—	55	123,462	8.9
11	WYB21	—	57	16,796	1,184	135,431	—	—	53	8	153,529	11.1
12	WYB71	—	83	6,529	16,276	109,143	30	70	—	10,165	142,296	10.2
	Total	15	1,386	25,562	639,453	510,617	45	70	53	188,749	1,382,706	
	Percentage (%) of ASVs	0.001	0.1	1.8	46.2	36.9	0.003	0.005	0.003	13.6		100

The differences between visually healthy and unhealthy seed potato for sample average of filtered reads, number of Firmicutes and number of Proteobacteria were statistically significant (*p* < 0.05) (Table 3). The number of filtered reads ranged from 131,118 to 251,553 (Table 1), and the number of ASVs ranged from 66,430 to 220,031 (Table 2). The mean number of filtered reads were 140,000 ± 9,000 (healthy samples) and 231,000 ± 19,000 (unhealthy samples). The difference of the mean values was statistically significant (*p* < 0.05). The number of ASVs from Firmicutes was 69,000 ± 34,000 in healthy samples and 7,000 ± 8,000 in unhealthy samples. Again, the difference of the mean values was statistically significant (*p* < 0.05). Similarly, the number of ASVs from Proteobacteria were 18,000 ± 16,000 (healthy) and 115,000 ± 18,000 (unhealthy), which was also statistically significant (*p* < 0.05) (Table 3).

**Table 3:** Comparison of unhealthy and healthy seed potato samples (mean ± SD) using Welch’s *t-*test

		**Healthy seed potato**	**Unhealthy seed potato**	**Student’s** ***t-*** **test** ***p*** **value**	**Difference**
1	Number of filtered reads	140,000 ± 9,000	231,000 ± 19,000	0.00914	Significant
2	Firmicutes	69,000 ± 34,000	7,000 ± 8,000	0.000564	Significant
3	Proteobacteria	18,000 ± 16,000	115,000 ± 18,000	0.003072	Significant
4	Number of ASVs	56,000 ± 31,000	102,000 ± 29,000	0.0812	Not significant

**Figure 2:**
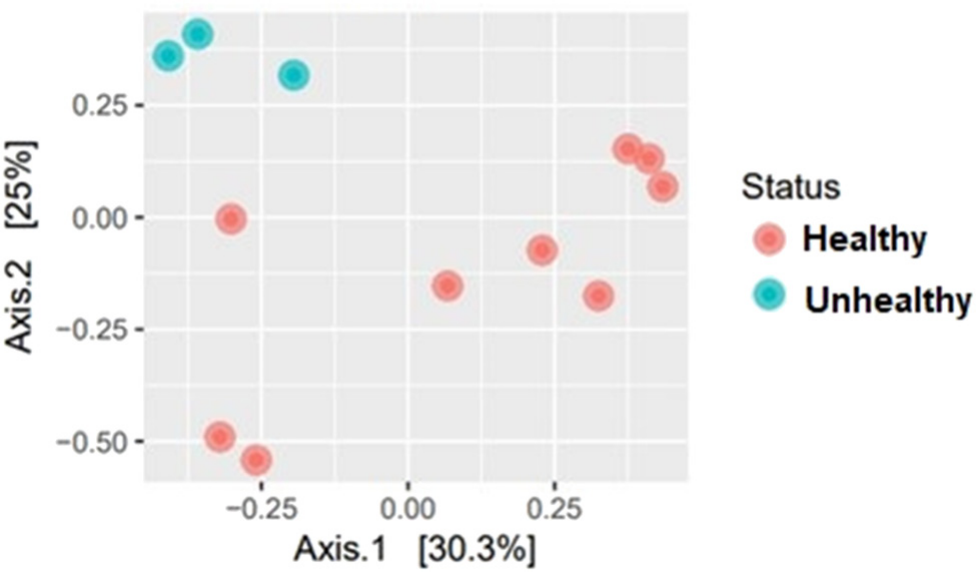
PCoA of the filtered data using the Bray–Curtis distance.

PCoA of the diversity of the microbial communities shows that the composition of the microbiota of the unhealthy tuber samples forms a separate cluster and is distinguished from the healthy samples (Figure 2).

### 3.2 Microbial diversity analysis

**Figure 3:**
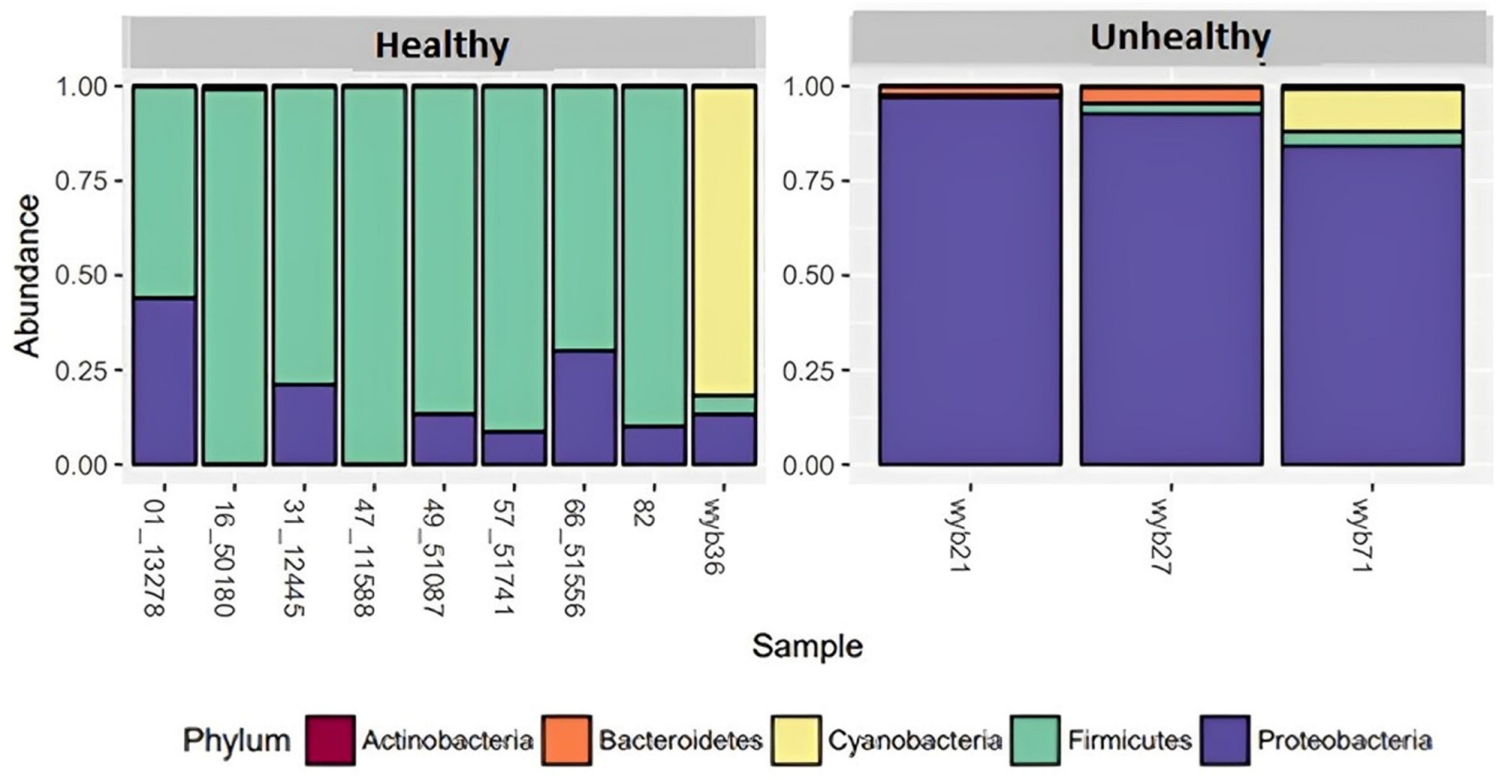
Overall relative abundance of microbial populations by phylum (mean abundance > 0.001).

Phylogenetic analysis revealed a diversity of microbes in the analysed seed potato. Bacteria belonging to eight phyla were identified: Acidobacteria, Actinobacteria, Bacteroidetes, Firmicutes, Proteobacteria, Saccharibacteria, Tenericutes, and Verrucomicrobiota (Figure 3). Sequences representing chloroplast DNA were categorized as belonging to cyanobacteria. Bacteria of these 8 phyla in turn belonged to 16 taxonomic classes, 29 bacterial orders, 55 families and 133 genera (Table S2).

Phylum Firmicutes was the most abundant bacteria in healthy potato (Figure 3). They were detected in all samples. However, there were differences in the availability of Firmicutes among the healthy and unhealthy tubers. The percentage of Firmicutes within visually healthy samples ranged from 55.9 to 99.9%, which establishes them as the dominant bacterial population within healthy tubers (Table 4). Conversely, in unhealthy tubers, Firmicutes varied from 0.5 to 3.9%, suggesting a clear association between the prevalence of Firmicutes and the health status of the seed potato (Table 4). Four taxonomic classes of Firmicutes were detected: Bacilli, Negativicutes, Clostridia, and Erysipelotrichia. In healthy tubers, only the taxonomic class Bacilli and taxonomic order Bacillales and six genera (*Bacillus, Salinibacillus, Staphylococcus, Lysinibacillus, Paenibacillus,* and *Brevibacillus*) were detected (Table S2). Bacteria belonging to these genera were not detected in unhealthy tubers.

**Table 4:** Proportion of ASVs identified in each of 12 seed potato samples

**Sample**		**Actinobacteria (%)**	**Bacteroidetes (%)**	**Firmicutes (%)**	**Proteobacteria (%)**	**Chloroplasts (%)**
1	01_13278	—	—	55.9	44	—
2	16_50180	—	—	98.9	0.1	0.8
3	31_12445	—	—	99.7	0.2	—
4	47_11588	—	—	99.8	0.1	—
5	49_51087	—	—	86.5	13.4	—
6	57_51741	—	—	91.2	8.7	—
7	66_51556	—	—	69.9	30	—
8	82	—	—	89.8	10.1	—
9	WYB36	—	—	4.9	13.3	81.6
10	WYB27	0.4	4	2.75	92.6	0.05
11	WYB21	—	2.3	0.5	97	0.03
12	WYB71	0.05	0.6	3.9	84.1	11.2

In unhealthy tubers, 20 genera of Firmicutes were detected, belonging to three taxonomic classes: Negativicutes, Clostridia, Erysipelotrichia, and Bacilli (Order Lactobacillales). They were *Enterococcus, Vagococcus, Carnobacterium, Anaerosinus, Pelosinus, Selenomonas, Anaerosporomusa, Sporomusa, Clostridium, Lachnoclostridium, Mobilitalea, Cellulosilyticum, Tyzzerella, Peptoclostridium, Caproiciproducens, Ruminiclostridium, Intestinimonas, Anaerotruncus,* and *Erysipelothrix* (Table S2).

Microbial populations of the rotten unhealthy tubers were dominated by the phylum Proteobacteria. Alpha, beta, gamma, delta, and epsilon proteobacteria were abundant in unhealthy tubers but some genera were also present in healthy tubers as well (at low abundances) (Table S1). Gamma proteobacteria was the taxonomic class with the highest abundance (28.85%) within unhealthy tubers (214 out of 745 detected ASVs).

*Aridibacter* sp. belonging to phylum Acidobacteria were detected in minute amounts only in one sample, which was visually healthy (Table 2). Acidobacteria is a phylum particularly abundant in soil habitats [16]. However, Acidobacteria represented only 0.001% of detected ASVs in seed potato.

Actinobacteria were detected only in unhealthy tubers (18 bacterial genera) but only at 0.1% of the total detected bacteria (Table 2). Similarly, the phylum Bacteroidetes (1.8%) was present in unhealthy tubers but not in any healthy tubers.

**Figure 4:**
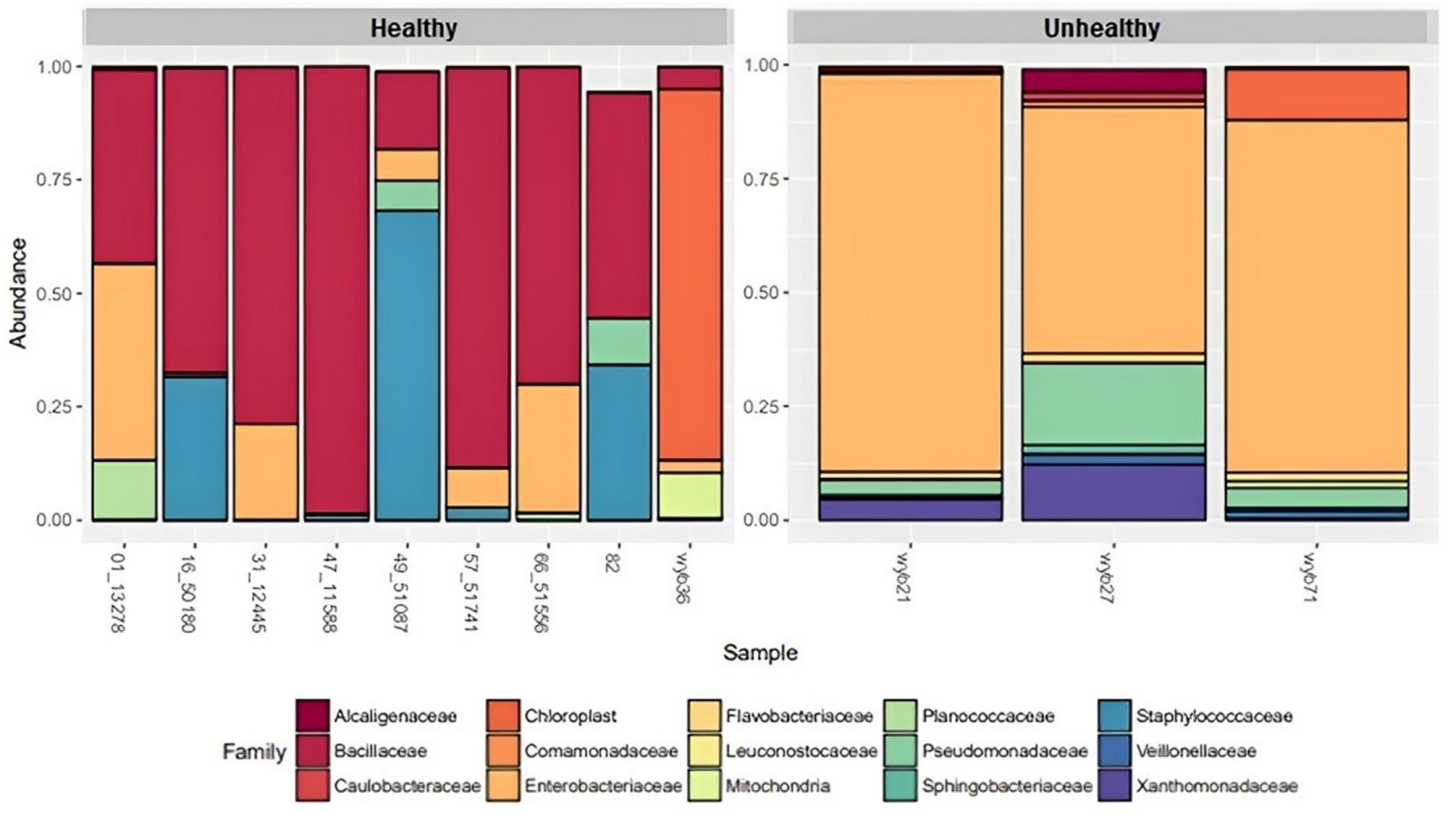
Overall relative abundance of microbial populations by family (mean abundance > 0.001).

Gamma-proteobacteria detected within the unhealthy tubers were the most diverse Proteobacteria with 150 different bacterial species (Table S2), which belonged to four families: Enterobacteriaceae, Pseudomonadaceae, Moraxellaceae, and Xanthomonadaceae (Figure 4).

**Figure 5:**
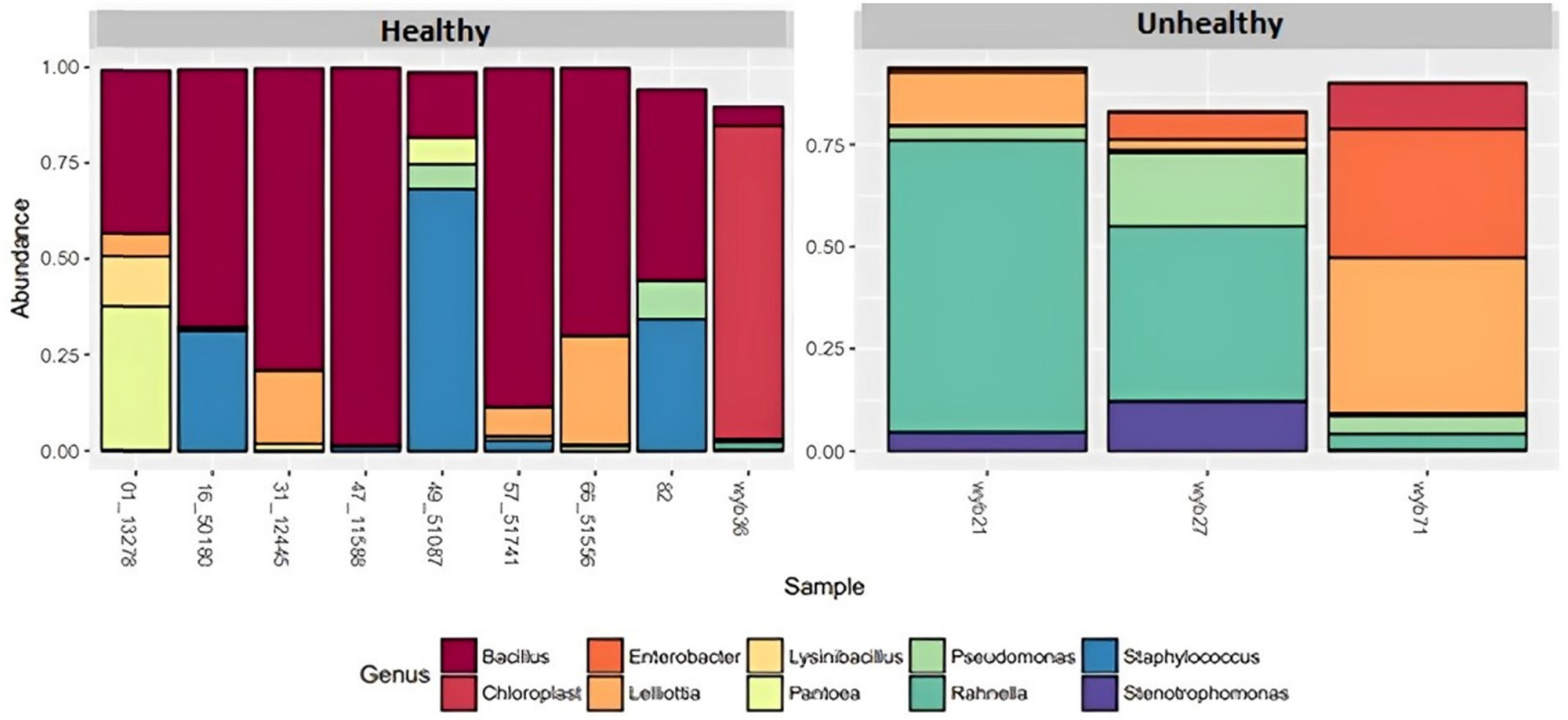
Overall relative abundance of microbial populations by Genus (mean abundance > 0.001).

The highly abundant gamma proteobacteria within unhealthy tubers were the *Rahnella* sp. belonging to the Enterobacteriaceae family (Figure 5). Lelliottia species belonging to Enterobacteriaceae family were detected both in healthy and unhealthy tubers (Figure 5). They are facultative anaerobes that have been identified in diverse natural environments, food, and water [17,18]. Certain Lelliottia species are suspected to have pathogenic possibilities [19].

Chloroplast DNA was categorized as cyanobacteria and was detected in minute quantities in all but one sample, where chloroplast DNA was abundant. However, chloroplasts are present in potato tubers as well [20] and chloroplast DNA originating from potato tubers cannot be disregarded. Potato tubers contain a large number of starch-storing amyloplasts and, upon exposure to light, amyloplasts in the peripheral cell layers develop into chloroplasts that are capable of photosynthesis [21].

## 4 Discussion

The results of this metagenomic analysis of seed potato endophytic bacteria revealed a comprehensive characterization of the microbial communities inhabiting the potato tissues both healthy and unhealthy. Paired-end sequencing of libraries using MiSeq Illumina technology generated a substantial amount of data, totalling 500 Mb. After quality filtering, which ensured the removal of low-quality reads, 2.05 million high-quality 16S rRNA gene sequence reads were retained. Subsequent removal of chimeric sequences resulted in a final dataset of 1.38 million sequences distributed across the 12 samples analysed.

The majority of these sequences were identified as ASVs, highlighting the high resolution of the analysis. A total of 745 unique ASVs were identified, indicating a diverse array of endophytic bacterial taxa associated with seed potatoes. These ASVs were assigned to 745 different taxa spanning 7 taxonomic ranks. The analysis revealed that the endophytic bacterial communities associated with seed potatoes were predominantly composed of taxa belonging to the phyla Firmicutes (46.2%) and Proteobacteria (36.9%). Additionally, we observed contributions from chloroplasts (13.6%), Bacteroidetes (1.8%), Actinobacteria (0.1%), Tenericutes (0.005%), Saccharibacteria (0.003%), Verrucomicrobiota (0.003%), and Acidobacteria (0.001%). The abundance and diversity of these bacterial taxa reflect the complex microbial ecosystem within seed potatoes. The dominance of Firmicutes and Proteobacteria suggests their potential importance in the endophytic bacterial community associated with potato tissues. Furthermore, the presence of chloroplasts underscores the importance of considering plant-associated contaminants in metagenomic analyses. The average number of filtered reads and non-chimeric sequences obtained from each sample provided insights into the variability of endophytic bacterial communities among seed potato samples. Additionally, the average amplicon size of 604 bp reflects the robustness of our sequencing approach in capturing diverse microbial taxa present in the potato tissues. Further, seed potato samples were collected through random sampling and the collected 12 samples included 9 healthy and 3 unhealthy samples, which reflects the actual distribution of healthy and unhealthy samples in the population. Despite a limited number of samples, meaningful insights can still be obtained from metagenomic analysis. This approach focused on capturing the diversity of microbial communities rather than the sheer sample size. The depth of sequencing and the accuracy in sequence analysis were often considered as more crucial than the number of samples analysed.

A comparison between visually healthy and unhealthy seed potatoes revealed significant differences in several key parameters, shedding light on the microbial dynamics associated with potato health status. The analysis demonstrated statistically significant variations in the average number of filtered reads, as well as the abundance of Firmicutes and Proteobacteria, between healthy and unhealthy seed potato samples. The observed differences in the number of filtered reads between visually healthy and unhealthy seed potatoes highlight potential differences in the microbial load and diversity associated with the two conditions. Visually unhealthy seed potatoes exhibited a significantly higher average number of filtered reads compared to healthy samples, suggesting a potential association between microbial abundance and potato health status. The PCoA conducted to assess the diversity of microbial communities associated with healthy and unhealthy tuber samples also revealed distinct clustering patterns, indicating significant differences in the composition and structure of bacterial communities between the two conditions. The separation of unhealthy tuber samples into a distinct cluster from healthy samples on the PCoA plot underscores the profound impact of tuber health status on the microbial community composition. Also, this finding underscores the importance of considering microbial community dynamics in the context of potato health and diseases.

The data show that the DNA concentration, the number of sequences, and the number of microbes detected within the samples vary depending on the healthy and unhealthy nature of the samples (Table 2). The large difference in the DNA concentration between healthy and unhealthy seed potato microbial DNA samples could be attributed to factors such as microbial load and microbial composition.

A large difference in the number of non-chimeric sequences between healthy and unhealthy seed potato samples was also observed. The number of ASVs from Firmicutes was 69,000 ± 34,000 in healthy samples and 7,000 ± 8,000 in unhealthy samples, showing a significant difference. Certain pathogens or stress conditions associated with unhealthy seed potatoes could drive more consistent changes in the microbial community composition across samples, which might result in population shifts.

Moreover, our analysis revealed significant disparities in the abundance of specific bacterial taxa, particularly Firmicutes and Proteobacteria, between healthy and unhealthy seed potato samples. Healthy seed potatoes exhibited a notably higher number of ASVs from Firmicutes compared to unhealthy samples, indicating a potential role of Firmicutes in maintaining potato health. Conversely, unhealthy seed potatoes showed a significantly higher abundance of ASVs from Proteobacteria, suggesting a potential association between Proteobacteria and potato disease or stress conditions.

The observed differences in the abundance of Firmicutes and Proteobacteria between healthy and unhealthy seed potatoes underscore the complex interactions between microbial communities and plant health. Firmicutes have been reported to include beneficial bacteria known for their plant growth-promoting properties and ability to suppress pathogens, which may contribute to the observed association with healthy seed potatoes. Garbeva et al. [22] detected Firmicutes within potato tubers by using a culture-based approach. In cassava, the dominant phylum across all tuber samples was Firmicutes (Ha et al. [23]). In addition, Firmicutes have been detected at lower abundance in diseased rhizosphere soil than in healthy rhizosphere soil (Lee et al. [24]). Consistent with all of the above findings, this study identified Firmicutes as a common dominant phylum in healthy potato tubers. The Firmicutes detected within the healthy tubers included various *Bacillus* species (Table S2). *Bacillus subtilis* can protect against potato common scab caused by *Streptomyces* sp. (Wang et al. [25]; Zhou et al. [26]). *Bacillus amyloliquefaciens* inhibits the growth and sporulation of *S. scabies* and secrete secondary metabolites against *S. scabies* (Lin et al. [27]). *Bacillus velezensis* inhibits five potato pathogens: *Streptomyces galilaeus, Phoma foveata, Rhizoctonia solani, Fusarium avenaceum* and *Colletotrichum coccodes* (Cui et al. [28]). The *Bacillus* species detected within the healthy tubers might therefore be protecting against tuber decay and pathogen invasion, therefore promoting tuber health; however, this would need to be confirmed by further research.

In contrast, the higher abundance of Proteobacteria in unhealthy seed potatoes may reflect a shift towards opportunistic or pathogenic taxa in response to stress or disease conditions. Within the domain bacteria, the phylum proteobacteria constitutes at present the largest and phenotypically most diverse phylogenetic lineage (Kersters et al. [29]). The highest diversity in bacteria belonging to phylum Proteobacteria was observed in visually unhealthy tuber samples, which contained 64 genera (alpha – 11, beta – 23, gamma – 26, delta – 3, epsilon – 1) (Table S2). Proteobacteria has been reported to harbour a large group of metabolic enzymes, which have a strong influence on global nitrogen and carbon cycles, as well as soil metabolites due to their great metabolic diversity. These pathogenic bacteria secrete proteins to adhere to and degrade plant cell walls, suppress plant defence responses, and deliver bacterial DNA and proteins into the cytoplasm of plant cells (Preston et al. [30]). Gammaproteobacterial diversity and community members have been identified as potential health indicators (Köberl et al. [31]). Healthy plants have been associated with an increase in potentially beneficial *Pseudomonas* and *Stenotrophomonas* species, while diseased plants have been associated with *Enterobacteriaceae*, known for their plant-degrading capacity (Köberl et al. [31]).

In addition, *Aridibacter* sp., a member of the Acidobacteria phylum, was detected in small amounts in only one visually healthy sample. Acidobacteria are typically abundant in soil habitats, and their detection in seed potato tubers is noteworthy, although at extremely low abundance. The limited presence of Acidobacteria in seed potatoes suggests that these bacteria may play a minor role in the endophytic microbial community of potatoes, despite their prevalence in soil environments. Similarly, Actinobacteria were exclusively detected in unhealthy tubers, although comprising only 0.1% of the total detected bacteria. The phylum Bacteroidetes was present solely in unhealthy tubers, at a relatively low abundance of 1.8%. *Flavobacterium* sp. have been identified as tuber microbiota responsible for potato tuber storage stability. They influence sprouting behaviour by inhibiting potato bud outgrowth (Buchholz et al. [32]). In this study, *Flavobacterium lindanitolerans* belonging to the Bacteroidetes phylum were detected in the unhealthy tubers. These findings suggest that certain bacterial taxa may be associated with tuber health status, potentially indicating their involvement in pathogenic or stress-related processes.

The detection of chloroplast DNA, categorized as cyanobacterial, in small quantities across most samples, with one sample exhibiting abundant chloroplast DNA, poses interesting considerations. While chloroplasts are naturally present in potato tubers, the detection of chloroplast DNA may reflect the presence of intact chloroplasts or remnants of chloroplast DNA from potato tissues. The development of chloroplasts in response to light exposure in peripheral cell layers of tubers further underscores the dynamic nature of chloroplast presence in potato tissues.

Of particular interest is the diversity and abundance of gamma-proteobacteria within unhealthy tubers, comprising 150 different bacterial species belonging to four families. *Rahnella* sp., a member of the Enterobacteriaceae family, was notably abundant within unhealthy tubers. The presence of diverse gamma-proteobacteria, including potential pathogens such as *Rahnella* sp., highlights the complex microbial interactions occurring within unhealthy tubers and their potential implications for tuber health and disease. A similar observation has been reported for onion, where Rahnella strains were abundant (17%) in unhealthy onion bulbs and only 1% in healthy onion bulbs. However, they are recognized for producing indole compounds that can promote plant growth (Da Costa et al. [33]).

Furthermore, the detection of Lelliottia species, belonging to the Enterobacteriaceae family, in both healthy and unhealthy tubers raises intriguing questions about their roles and interactions within the potato endophytic microbiome. While Lelliottia species are commonly found in diverse natural environments, food, and water, certain strains have been implicated in pathogenicity. The detection of Lelliottia species in both healthy and unhealthy tubers underscores the need for further investigation into their potential roles in potato health and disease.

These findings from the metagenomic analysis provide compelling evidence of the association between tuber health status and the diversity and structure of microbial communities. Understanding the dynamics of microbial communities in relation to tuber health is crucial for elucidating the mechanisms underlying potato diseases, storage conditions and developing effective management strategies. The distinct clustering of unhealthy tuber samples may be attributed to various factors, including changes in environmental conditions within the tuber microenvironment, alterations in host plant physiology, and interactions with pathogenic microorganisms. Unhealthy tubers may create niche environments that select for specific microbial taxa capable of colonizing and thriving under conditions of stress or disease. Conversely, healthy tubers may harbour microbial communities associated with symbiotic or beneficial interactions that promote tuber health and resilience against pathogens.

Further investigations into the specific microbial taxa driving the observed differences between healthy and unhealthy tuber samples, as well as their functional roles in tuber health and disease, are warranted. Integration of metagenomic, metatranscriptomic, and metabolomic approaches will enhance our understanding of the complex interactions between microbial communities and potato health, ultimately facilitating the development of targeted interventions for disease management and sustainable potato production.

The endophytic bacteria present in the seed tubers may play an important role in seed piece decay, tuberization, and plant growth. In addition, pathogen infection had a greater impact on the bacterial population than the plant genotype [34]. Knowledge of plant-associated bacteria is essential not only for understanding their ecological role and their interaction with plants, but also for future biotechnological application. Recently, it has been demonstrated that bacterial endophytes may have beneficial effects on host plants, such as growth promotion and biological control of pathogens [35]. This study suggests that these bacteria detected in healthy tubers might interact more closely with the host plant and therefore could be efficient biological control agents in sustainable crop production.

Overall, these findings contribute to a better understanding of the taxonomic composition and diversity of seed potato endophytic bacteria, laying the foundation for future studies investigating their functional roles and potential applications in sustainable agriculture. In addition, this analysis provides valuable insights into the microbial dynamics associated with seed potato health status and highlight the potential role of specific bacterial taxa in influencing potato health and productivity. Future studies exploring the functional roles of these microbial communities and their interactions with host plants will further elucidate the mechanisms underlying plant–microbe interactions in agricultural systems, ultimately contributing to the development of strategies for disease management and sustainable crop production.

## 5 Conclusions

In this study, the diversity of endogenous bacterial communities associated with seed potato tubers was analysed. When compared with the visually healthy tubers, the unhealthy tubers showed marked changes in the endophytic bacterial community: (1) A shift from a Firmicutes-rich community to a Proteobacteria-rich community and (2) increased bacterial diversity.

The potato microbiota is mostly composed of four dominant bacterial phyla: Actinobacteria, Bacteroidetes, Firmicutes, and Proteobacteria. Firmicutes and Proteobacteria represent more than 90% of the total community of healthy and unhealthy tubers, respectively. Other subdominant phyla include Acidobacteria, Sacharibacteria, and Tenericutes. When transitioning from healthy to unhealthy, Proteobacteria populations outcompete Firmicute populations. While the implications and functions of these microbial populations are not fully clear, alteration of microbiota from Firmicutes to Proteobacteria-rich populations negatively impacts potato tuber health and can be used as an indicator.

## Supplementary Material

supplementary material
