# Supplementary material for: Metagenomic analysis of endophytic bacteria in seed potato (Solanum tuberosum)
Source: Open Life Sci. 2024 Jul 24;19(1):20220897. doi: 10.1515/biol-2022-0897 (PMC11282915; doi:10.1515/biol-2022-0897)
Supplement: supplementary material [file biol-2022-0897-sm.pdf]

Supplementary material

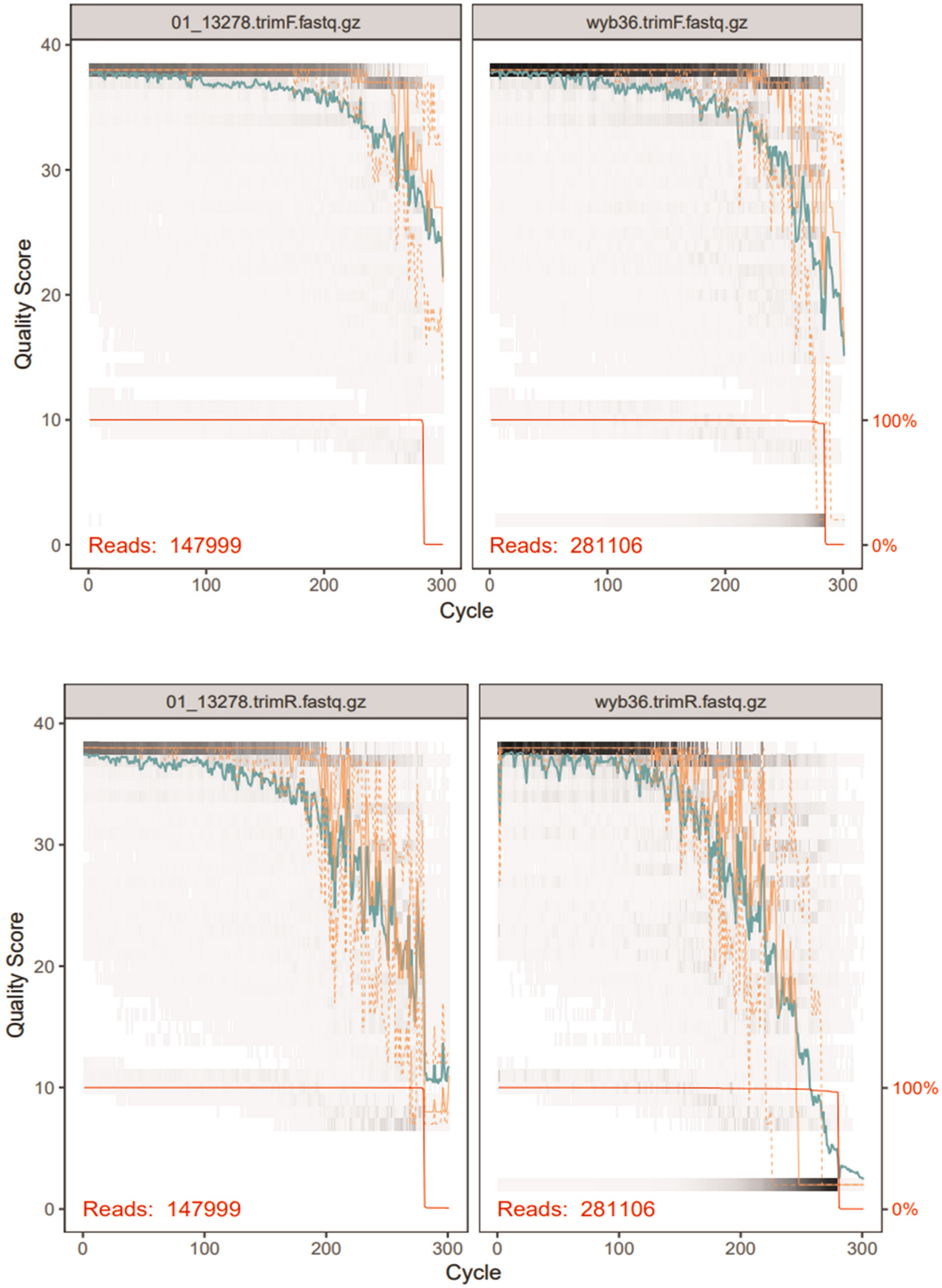

Figure S1. : Quality profiles of the forward and reverse reads for DNA extracted from two healthy potato tuber samples.

Table S2:

	Phylum	Class	Order	Family	Genus	Health status of the tuber
**1.**	Acidobacteria	Blastocatellia	Blastocatellales	Blastocatellaceae	Aridibacter	Unhealthy
**2.**	Actinobacteria	Actinobacteria	Micrococcales	Micrococcaceae	Glutamicibacter	Unhealthy
					Micrococcus	Unhealthy
					Rothia	Unhealthy
					Paenarthrobacter	Unhealthy
					Pseudarthrobacter	Unhealthy
					Sinomonas	Unhealthy
				Microbacteriaceae	Microbacterium	Unhealthy
				Dermabacteraceae	Brachybacterium	Unhealthy
				Brevibacteriaceae	Brevibacterium	Unhealthy
				Intrasporangiaceae	Knoellia	Unhealthy
				Ruaniaceae	Haloactinobacterium	Unhealthy
				Cellulomonadaceae	Oerskovia	Unhealthy
				Micrococcales_	Luteimicrobium	Unhealthy
				Sanguibacteraceae	Sanguibacter	Unhealthy
			Corynebacteriales	Corynebacteriaceae	Corynebacterium	Unhealthy
				Nocardiaceae	Rhodococcus	Unhealthy
			Micromonosporales	Micromonosporaceae	Dactylosporangium	Unhealthy
			Propionibacteriales	Propionibacteriaceae	Propionibacterium	Unhealthy
**3.**	Bacteroidetes	Bacteroidia	Bacteroidales	Bacteroidaceae	Bacteroides	Unhealthy
				Porphyromonadaceae	Dysgonomonas	Unhealthy
		Cytophagia	Cytophagales	Cytophagaceae	NA	Unhealthy
		Flavobacteriia	Flavobacteriales	Flavobacteriaceae	Flavobacterium	Unhealthy
					Myroides	Unhealthy
					Chryseobacterium	Unhealthy
					Empedobacter	Unhealthy
					Epilithonimonas	Unhealthy
					Moheibacter	Unhealthy
		Sphingobacteriia	Sphingobacteriales	Sphingobacteriaceae	Sphingobacterium	Unhealthy
					Pedobacter	Unhealthy
					Olivibacter	Unhealthy
					Arcticibacter	Unhealthy
				Chitinophagaceae	Taibaiella	Unhealthy
					Vibrionimonas	Unhealthy
					Sediminibacterium	Unhealthy
**4.**	**Cyanobacteria**	**Chloroplast**	**Chloroplast**	**Chloroplast**		
**5.**	Firmicutes	Bacilli	Bacillales	Bacillaceae	Bacillus	Healthy
					Salinibacillus	Healthy
				Staphylococcaceae	Staphylococcus	Healthy

(Continued)

Table S2: *Continued*

	Phylum	Class	Order	Family	Genus	Health status of the tuber
				Planococcaceae	Lysinibacillus	Healthy
				Paenibacillaceae	Paenibacillus	Healthy
					Brevibacillus	Healthy
			Lactobacillales	Leuconostocaceae	Leuconostoc	Unhealthy & Healthy
				Enterococcaceae	Enterococcus	
					Vagococcus	Unhealthy
				Carnobacteriaceae	Carnobacterium	Unhealthy
		Negativicutes	Selenomonadales	Veillonellaceae	Anaerosinus	Unhealthy
					Pelosinus	Unhealthy
					Selenomonas	Unhealthy
					Anaerosporomusa	Unhealthy
					Sporomusa	Unhealthy
		Clostridia	Clostridiales	Clostridiaceae	Clostridium_sensu_stricto	Unhealthy
				Lachnospiraceae	Lachnoclostridium	Unhealthy
					Mobilitalea	Unhealthy
					Cellulosilyticum	Unhealthy
					Tyzzerella	Unhealthy
				Peptostreptococcaceae	Peptoclostridium	Unhealthy
				Ruminococcaceae	Caproiciproducens	Unhealthy
					Ruminiclostridium_5	Unhealthy
					Intestinimonas	Unhealthy
					Anaerotruncus	Unhealthy
		Erysipelotrichia	Erysipelotrichales	Erysipelotrichaceae	Erysipelothrix	Unhealthy
**6.**	Proteobacteria	Alphaproteobacteria	Caulobacterales	Caulobacteraceae	Brevundimonas	Unhealthy & Healthy
					Asticcacaulis	Unhealthy
					Phenylobacterium	Unhealthy
					Caulobacter	Healthy
			Rickettsiales	Mitochondria	Mitochondria	
			Rhizobiales	Brucellaceae	Ochrobactrum	Unhealthy & Healthy
				Hyphomicrobiaceae	Devosia	Healthy
				Rhizobiaceae	Rhizobium	Healthy
				Methylobacteriaceae	Methylobacterium	Healthy
			Rhodobacterales	Rhodobacteraceae	Rubellimicrobium	Unhealthy & healthy
			Sphingomonadales	Sphingomonadaceae	Sphingopyxis	

(Continued)

Table S2: *Continued*

Phylum	Class	Order	Family	Genus	Health status of the tuber
					Unhealthy & healthy
		Rhodospirillales	Rhodospirillales_Incertae_Sedis	Reyranella	Healthy & Unhealthy
	Betaproteobacteria	Burkholderiales	Alcaligenaceae	Advenella	Unhealthy & healthy
				Achromobacter	Unhealthy & healthy
				Alcaligenes	Unhealthy & healthy
				Bordetella	Unhealthy
				Candidimonas	Unhealthy
				Verticia	Unhealthy & healthy
				Parapusillimonas	Unhealthy
			Comamonadaceae	Comamonas	Unhealthy & healthy
				Delftia	Unhealthy
				Variovorax	Unhealthy & healthy
				Curvibacter	Unhealthy & Healthy
				Hydrogenophaga	Unhealthy & healthy
				Aquabacterium	Healthy
				Pelomonas	Healthy
				Xenophilus	Unhealthy
				Kinneretia	Healthy
			Burkholderiaceae	Ralstonia	Unhealthy & healthy
				Burkholderia-Paraburkholderia	Unhealthy & healthy
			Oxalobacteraceae	Oxalicibacterium	Unhealthy
				Undibacterium	Healthy
				Herminiimonas	Unhealthy
				Massilia	Healthy
		Neisseriales	Neisseriaceae	Vogesella	Healthy
	Gamma proteobacteria	Enterobacteriales	Enterobacteriaceae	Lelliottia	

(Continued)

Table S2: *Continued*

Phylum	Class	Order	Family	Genus	Health status of the tuber
					Unhealthy & healthy
				Rahnella	Unhealthy & healthy
				Enterobacter	Unhealthy healthy
				Pantoea	Unhealthy & healthy
				Erwinia	Unhealthy
				Citrobacter	Unhealthy
				Salmonella	Unhealthy
				Raoultella	Unhealthy & healthy
				Serratia	Unhealthy
				Yersinia	Unhealthy
				Cronobacter	Unhealthy
				Morganella	Unhealthy
				Esherichica/Shigella	Healthy
				Kluyvera	
				Buttiauxella	
				Klebsiella	Unhealthy & healthy
				Kluyvera	Unhealthy
				Providencia	
				Budvicia	Unhealthy
				Buttiauxella	Unhealthy & healthy
				Proteus	Unhealthy
				Providencia	Unhealthy
			Pseudomonadaceae	Pseudomonas	Unhealthy & healthy
			Moraxellaceae	Acinetobacter	Unhealthy & healthy
				Enhydrobacter	Healthy
		Xanthomonadales	Xanthomonadaceae	Stenotrophomonas	Unhealthy & healthy
				Luteimonas	Unhealthy & healthy

(Continued)

Table S2: *Continued*

	Phylum	Class	Order	Family	Genus	Health status of the tuber
					Ignatzschineria	Unhealthy
					Dyella	Healthy
					Acidibacter	Healthy
					Pseudoxanthomonas	Unhealthy
				Nevskiaceae	Nevskia	Healthy
			Aeromonadales	Aeromonadaceae	Aeromonas	Healthy
			Acidiferrobacterales	Acidiferrobacteraceae	NA	Healthy
			Cellvibrionales	Cellvibrionaceae	Cellvibrio	Unhealthy & healthy
			Chromatiales	Chromatiaceae	Rheinheimera	Healthy
		Deltaproteobacteria	Myxococcales	NA	NA	Unhealthy & healthy
			Bdellovibrionales	Bacteriovoracaceae	Peredibacter	Unhealthy
			Desulfovibrionales	Desulfovibrionaceae	Desulfovibrio	Unhealthy
			Bradymonadales	Bradymonadaceae	Bradymonas	Unhealthy
		Epsilon proteobacteria	Campylobacterales	Campylobacteraceae	Arcobacter	Unhealthy
**7.**	Saccharibacteria	NA	NA	NA	NA	Unhealthy
**8.**	Tenericutes	Mollicutes	T2WK15B57	NA	NA	Unhealthy
